# Comparative Transcriptome Analysis of *Streptomyces Clavuligerus* in Response to Favorable and Restrictive Nutritional Conditions

**DOI:** 10.3390/antibiotics8030096

**Published:** 2019-07-19

**Authors:** Laura Pinilla, León F. Toro, Emma Laing, Juan Fernando Alzate, Rigoberto Ríos-Estepa

**Affiliations:** 1 Grupo de Bioprocesos, Universidad de Antioquia, Calle, Medellín 70 No. 52–21, Colombia; 2 Department of Microbial Sciences, School of Biosciences and Medicine, University of Surrey, Guildford GU2 7XH, UK; 3 Group of Parasitology, School of Medicine, Universidad de Antioquia, Calle, Medellín 70 No. 52-21, Colombia; 4 Grupo de Bioprocesos, Departamento de Ingeniería Química, Universidad de Antioquia, Calle, Medellín 70 No. 52-21, Colombia

**Keywords:** transcriptomic analysis, *Streptomyces clavuligerus*, gene expression, clavulanic acid, complex media, gene cluster

## Abstract

*Background:* Clavulanic acid (CA), a β-lactamase inhibitor, is industrially produced by the fermentation of *Streptomyces clavuligerus*. The efficiency of CA production is associated with media composition, culture conditions and physiological and genetic strain characteristics. However, the molecular pathways that govern CA regulation in *S. clavuligerus* remain unknown. *Methods and Results:* Here we used RNA-seq to perform a comparative transcriptome analysis of *S. clavuligerus* ATCC 27064 wild-type strain grown in both a favorable soybean-based medium and in limited media conditions to further contribute to the understanding of *S. clavuligerus* metabolism and its regulation. A total of 350 genes were found to be differentially expressed between conditions; 245 genes were up-regulated in favorable conditions compared to unfavorable. *Conclusion:* The up-regulated expression of many regulatory and biosynthetic CA genes was positively associated with the favorable complex media condition along with pleiotropic regulators, including proteases and some genes whose biological function have not been previously reported. Knowledge from differences between transcriptomes from complex/defined media represents an advance in the understanding of regulatory paths involved in *S. clavuligerus’* metabolic response, enabling the rational design of future experiments.

## 1. Introduction

Clavulanic acid (CA) is a potent β-lactamase inhibitor; it increases the stability and half-life of β-lactam antibiotics [1]. All CA clinically used is obtained by fermentation of *Streptomyces clavuligerus* species; this actinomycete is a producer of a large variety of secondary metabolites with different biological activities e.g., cephamycin C (cephC) and various compounds with clavam structure with antifungal and antitumor activities [2]. 

**Figure 1:**
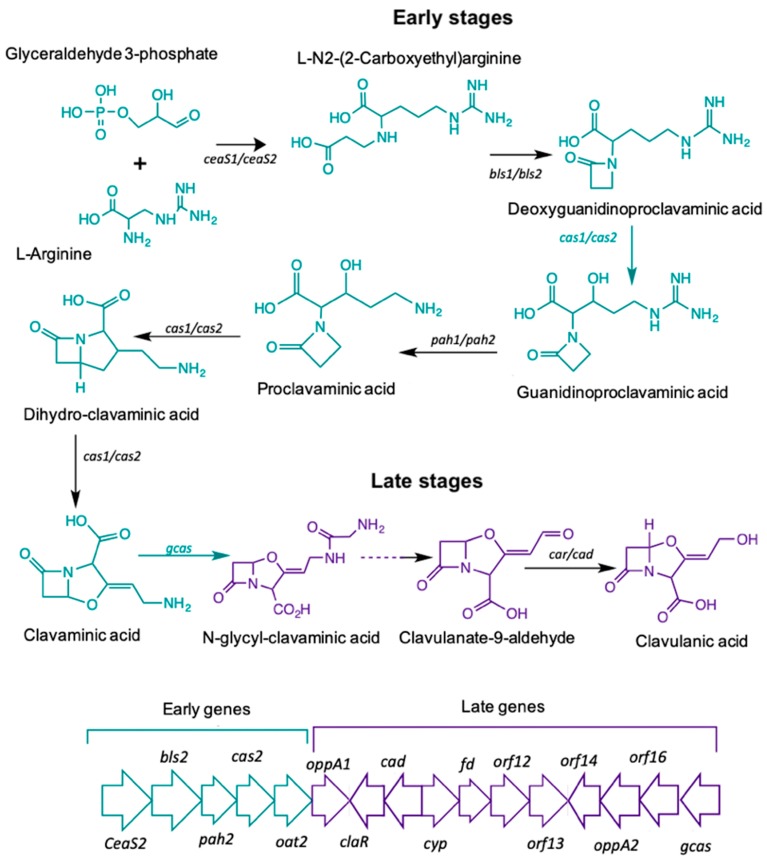
Clavulanic acid metabolic pathway.

The biosynthetic pathway leading to CA starts with the condensation of the amino acid L-arginine (precursor C_5_), and the glycolytic intermediate glyceraldehyde-3-phosphate (GAP), (precursor C_3_). The reaction is mediated by N_2_-(2-carboxyethyl)-arginine synthetase (*CeaS*) producing the acyclic compound N_2_-(2-carboxyethyl) arginine, which, after successive oxidation-reduction reactions, gives rise to clavaminic acid, which differs from CA in its stereochemistry [3]. The conversion of clavaminic acid to CA requires the inversion of the stereochemistry of the ring system to form CA. The structural similarities between CA and 5S clavams reflect shared elements of a common biosynthetic pathway. The steps shared in the biosynthetic pathways are called "early stages", which lead to the last common intermediate, clavaminic acid. The specific stages, used for the production of CA or 5S clavams, are called "late stages" (Figure 1) [4].

The production of secondary metabolites in *Streptomyces* sp. depends, in part, on growth and morphological differentiation; these processes are triggered in response to nutrient shortage, mainly during the stationary phase [3,5,6]. *S. clavuligerus* also presents a complex life cycle in which germinative spores develop giving rise to a multinucleated mycelial substrate that later becomes aerial mycelium; this initiates a process of regular septation along with the aerial hyphae, ending in uninucleate long chain spores that, once separated, lead to germinative spores that initiate the life cycle again. After initiation of the morphological differentiation process, secondary metabolites generally begin to be synthesized. The expression of genes, required for these two events, are regulated in a coordinated manner [7].

Despite numerous studies, the regulatory mechanisms present in the CA biosynthesis pathway are yet to be identified. CA production is controlled by a group of regulatory genes; however, most studies have focused on individual genes. In order to develop a rational genetic engineering approach for secondary metabolite production it is necessary to use modern methods to obtain information on the temporal and conditional expression of global regulatory factors, specific-pathway regulators and limiting enzyme production rates. Hence, the application of transcriptomics to *S. clavuligerus,* growing at different stages of cell development and/or environmental conditions, might contribute to elucidate regulatory paths [8,9,10,11].

Due to the clinical and industrial interest of CA, most studies on *S. clavuligerus* have been directed to increase its production titers, evaluating the impact of culture media composition and environmental conditions [6,12], metabolic engineering [13], transcriptional analysis (microarrays) and recently, metabolic network and systems biology analysis [14,15,16,17,18]. Recently, the use of data from RNA-seq experiments has greatly contributed to increase our understanding of regulatory networks in diverse microorganisms. Transcriptomics enables quantitative measurements of mRNA expression and variations between different states, thus reflecting the genes that are being overexpressed or downregulated at particular times and conditions [11]. The main goal of these experiments is to identify the differentially expressed genes in two or more conditions selected based on a combination of expression change threshold and score cutoff, which are usually based on P values generated by statistical modeling [19].

Data from RNA-Seq have proved to be particularly useful in the study of cell metabolic performance through the study of transcriptional regulators and differential gene expression analysis [11], thus contributing to strain optimization studies, using metabolic engineering tools. As a result, a better understanding of the metabolic pathways and regulatory mechanisms involved in the biosynthesis of important metabolites, such as CA, has been gained. 

In this study, we performed a comparative transcriptome analysis of *S. clavuligerus* subjected to two culture conditions, growing cells in a nutrient-deficient medium, unfavorable for CA biosynthesis, and in nutrient conditions favorable for CA biosynthesis, using a soybean-based medium. The study aimed to contribute to further understanding of secondary metabolism and potential regulatory mechanisms that might be involved in CA synthesis.

## 2. Results

### 2.1. Shake-Flask Cultivations Under Different Culture Conditions

**Figure 2:**
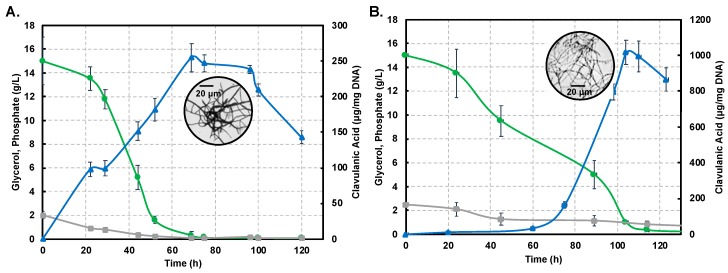
Dynamics of substrate consumption and product biosynthesis in flask cultures of *Streptomyces clavuligerus* ATCC 27064. (**A**) Chemically-defined media GSPG. (**B**). Soybean protein isolate media SB-M. Color code: Clavulanic acid (blue), glycerol (green), phosphate (grey). Note that the secondary axis depicting CA production shows differences in scale, associated with each culture medium.

Figure 2A,B show profiles for substrate consumption and product biosynthesis using the *S. clavuligerus* ATCC27064 strain, grown in minimal GSPG and soybean-based SB-M media, respectively.

As observed, the phosphate source was completely depleted at 75 h in GSPG; meanwhile, in SB-M, the phosphate consumption was slower, rendering a residual concentration of 0.82 g/L at 114 h. The main carbon source, glycerol, was completely consumed at 75 h in GSPG, in contrast to SB-M for which the lowest concentration was obtained at 114 h; 0.08 g/L of glycerol remained unused in SB-M due to the presence of alternative carbon sources such as starch, peptides and carbohydrates in isolated soybean protein [20,21]. CA production was considerably higher in SB-M (1015.16 μgCA/mgDNA) at 104 h compared to the maximum CA reached in GSPG (238.37 μgCA/mgDNA) at 72 h.

Differences in strain metabolic response to nutrient perturbation were confirmed not only by CA titers but also by growth measured by the indirect DNA technique. The maximum DNA concentration in biomass produced in the GSPG medium was 97 µg DNA/mL at 22 h; in SB-M the higher concentration was reached at 24 h (153 µg DNA/mL). Circles in Figure 2A,B show the morphology associated with maximum CA concentration in the GSPG and SB-M conditions, respectively. In both conditions, after 75 h of culture, the disintegration of pellet-like morphology was observed giving rise to disperse hyphae with no branches [22]; the starting point of pellet structure lysis and appearance of non-branched dispersed hyphae was observed, which continued until 120 h. However, in the SB-M condition, the hyphae presented separate compartments, reflective of defective (incomplete) sporulation, and the concomitant decrease in cellular concentration, characteristic of the death phase [23].

### 2.2. Identification of Differentially Expressed Genes Between SB-M and GSPG Conditions

RNA-Seq data analysis revealed a small group of differentially expressed genes; 245 genes were up-regulated in SB-M and 105 were up-regulated in GSPG. The 350 differentially expressed genes constitute 4.73% of the total genes in the *S. clavuligerus* genome (Supplementary Table S1). A large percentage of these genes are located in the plasmid pSCL4 in *S. clavuligerus* (29% and 22% of total genes in plasmid were up-regulated in SB-M and GSPG, respectively). Differentially expressed genes involved in the main metabolic pathways (primary and secondary metabolism) and some regulated genes of *S. clavuligerus* are listed in Table 1.

Most of the genes in the CA cluster showed a higher expression when *S. clavuligerus* was cultivated in complex medium; cultivation time was 104 hours for which maximum CA production was achieved and RNA extraction was carried out (see Figure 2B). It was observed that the specific pathway CA regulator, *claR* (SCLAV_4181), along with the late coding genes N-glycyl-clavaminic acid synthetase *gcas* (SCLAV_4181) and clavulanate-9-aldehyde reductase *cad*/*car* (SCLAV_4190), were up regulated; the latter two are involved in the last step of the CA biosynthetic pathway (see Figure 1). Similarly, the global regulator *ccaR* (SCLAV_4204) was positively regulated for the SB-M condition. The *ceaS2* promoter directs the orchestrated transcription of the early CA biosynthesis genes *ceaS2*, *bls2*, *pah2* and *cas2*, not regulated by *claR*; *ccaR* is a controller when it binds to the bidirectional promoters of *cefD*, *cmcI* and *lat* in the cephamycin C gene cluster, triggering the expression of these operons [24,25].

Our results showed differential expression of malate dehydrogenase *mdh* and SCLAV_4122 in SB-M, thus contributing to the formation of oxaloacetate from malate and/or acetyl-CoA; both oxaloacetate and acetyl-CoA play an important role in the synthesis of amino acid anabolic precursors e.g., aspartate and lysine, for CA and CephC biosynthesis, respectively. Likewise, the formation of fumarate through succinate dehydrogenase (SCLAV_3769 and SCLAV_3770) might be an important precursor of argininosuccinate via *argH* (argininosuccinate lyase), whose expression also increased in SB-M. TCA-cycle-associated genes e.g., acyl-CoA dehydrogenase (SCLAV_0665), malate dehydrogenase (SCLAV_3742) and central metabolism-associated genes e.g., those coding for pyruvate dehydrogenase (SCLAV_1401) and aldehyde dehydrogenase (SCLAV_5677), were differentially expressed.

Arginine metabolism has also been extensively studied in *S. clavuligerus* since, together with GAP, they are direct precursors of CA. In our results, *oat2,* located in the CA cluster, along with *argJ,* had a higher expression in SB-M, whereas *oat1* expression was favored in GSPG. *oat1* and *oat2* are ornithine acetyltransferases, which transfer an acetyl group from N-acetylornithine to glutamate, increasing the pool of arginine via glutamate. Also, a differential expression of the genes associated with nitrogen transport was observed e.g., oligopeptide permeases (SCLAV_4407 and 4408), peptide transporters (SCLAV_p1156), the PII regulatory protein for nitrogen metabolism (SCLAV_4535), the ammonium transporter (SCLAV_4534) along with glutamine synthase (SCLAV0834) and N-acetylglutamate synthase (NAGS) (SCLAV_2388); the latter is involved in the metabolism of arginine and the urea cycle inter-connection. *oat1,* located in the clavam 5S cluster, has been reported as non-essential for CA (or clavams) production, but its role in some unidentified steps in CA-5S clavam biosynthetic pathway cannot be ruled out [26,27]. Furthermore, several genes related to growth or primary metabolism were up-regulated in the rich medium, e.g., 11 genes for ribosomal proteins and the EF-Tu elongation factor, 12 genes for the subunits of the NADH quinone reductase, genes for ATP synthases and cytochrome oxidases or 5 genes for branched amino acids SCLAV_1197 to 1201.

Correspondingly, acetylglutamate kinase, *argB*, N-acetyl-gamma-glutamyl-phosphate reductase, *argC* (2.52 FC), acetylornithine aminotransferase, *argD* (3.0 FC), arginine biosynthesis bifunctional protein and argininosuccinate synthase (SCLAV_0799) showed higher expression in the SB-M condition. Glutamate is an important amino acid precursor in CA biosynthesis [28,29]; glutamate conversion to arginine, reaction mediated by NAGS, involves ornithine as intermediate metabolite [28].

Further, for GSPG, most down-regulated genes are associated with cell maintenance and transport, as expected for a limited medium with low antibiotic concentrations. Down-regulated are three genes for alkaline peptidases, 4 genes for phenylacetic acid degradation and 4 genes for a putative lantibiotic (see Supplementary Table S1). Metabolism of phenylacetic acid has been described in other species of *Streptomyces* and phenylacetic acid has shown antifungal activities; thus, such activities cannot be ruled out for the case of *S. clavuligerus* [30]. Likewise, bacteriocins, peptide antibiotics produced by bacteria, e.g., lantibiotics, represent a rich repository that can yield a large number of valuable novel antimicrobials [31].

### 2.3. Validation of Differentially Expressed Genes by Quantitative qRT-PCR

**Figure 3:**
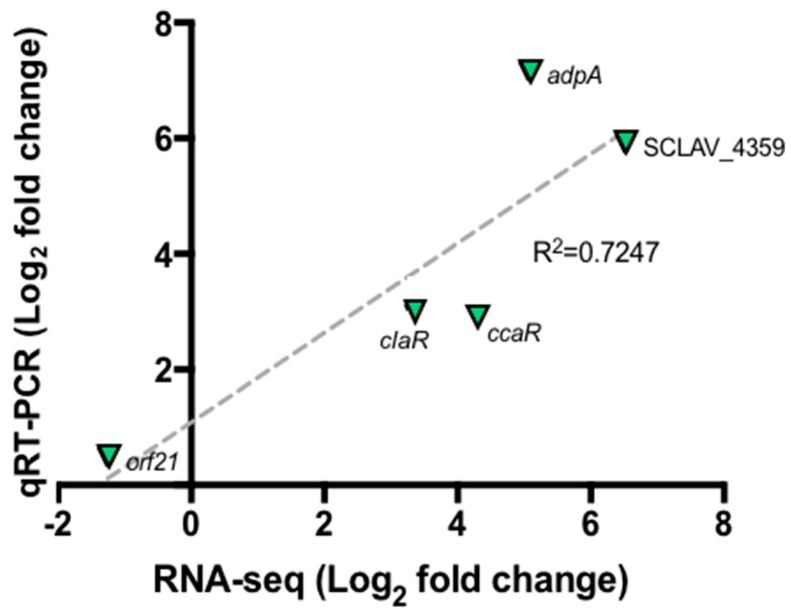
Quantitative real time PCR for selected genes *orf21*, *ccaR*, *adpA* and SCLAV_4359. Correlation between RNA-Seq experiment and qRT-PCR. The dashed gray line represents the least squares adjustment for the data points shown.

Figure 3 shows the relative expression of selected genes by taking the GSPG condition as control and SB-M as experimental condition so as to establish changes in gene expression when *S. clavuligerus* is grown under favorable conditions. Correlation analyses between RNA-seq and RT-qPCR data were performed using linear regression analysis (Pearson correlation). The results found by qRT-PCR for the selected genes are in concordance with those found by RNA-Seq; an acceptable correlation was obtained indicating the reliability of the RNA-seq analysis. Although qRT-PCR was used to verify expression differences, no protein analyses were performed to confirm that the differential expression was observed at the protein level. Some previous studies have shown poor correlation between RNA-Seq and proteomics analysis; the two approaches are best used in combination [32]. A good correlation (R^2^ = 0.51 to 0.98), considering mRNA expression levels and the protein levels, has been conveyed in the *Streptomyces* genus, using a proteomic analysis [33,34].

### 2.4. GO Term Enrichment Analysis

**Figure 4:**
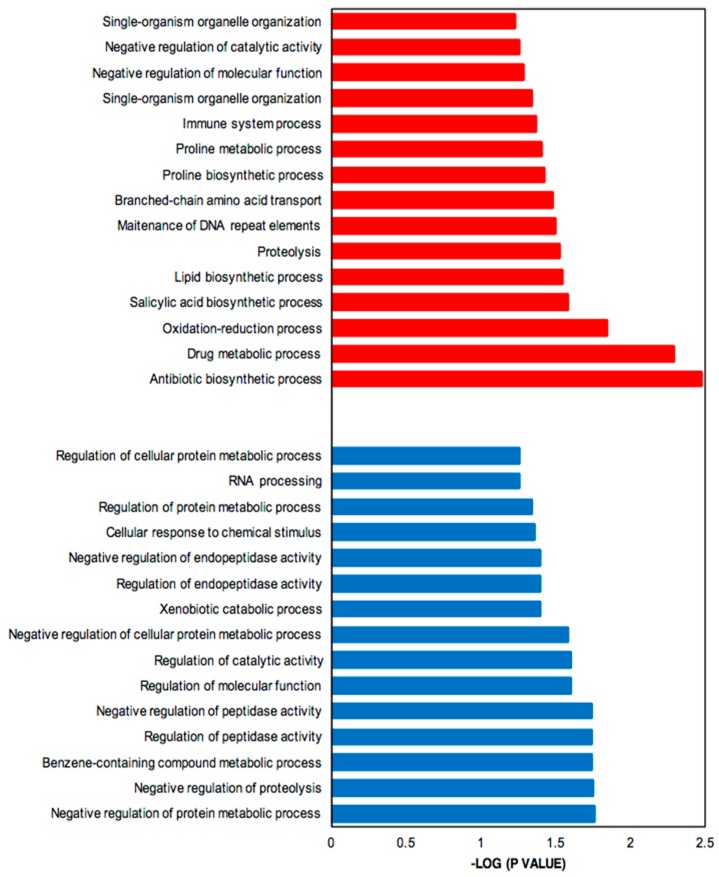
Bar plot for the −log10 of the *p*-value of selected GO terms (biological process). The up- and down-regulated genes (q-value (FDR) threshold) were subjected to gene ontology analysis using Ontologizer 2.0. The enriched biological processes were plotted. Red bar, enriched GO terms regulated by up-regulated genes; blue bar, enriched GO terms regulated by down-regulated genes.

Genes up- and down-regulated were subjected to functional enrichment analysis [35]. Figure 4 shows the significantly enriched Gene Ontology (GO) terms associated with up-regulated (more highly expressed in SB-M medium) (red) and down-regulated (more highly expressed in GSPG medium) genes (blue). The parent–child GO term analysis detected a total of 123 GO terms significantly overrepresented for the SB-condition and 46 for GSPG, both with a *p*-value < 0.01; from these, 36% refer to biological processes.

The up-regulated genes, which include genes involved in CA and CephC metabolism, were found to be related to antibiotic biosynthetic processes (GO:0017000) and drug metabolic processes (GO:0017144). Moreover, oxidation-reduction (GO: 0055114) was significantly enriched, in keeping with oxidoreductase acting on oxidative phosphorylation and control of oxidative stress. The complex medium also favors protein degradation, proteolysis (GO:0006508) and amino acid metabolism, mainly proline related-processes; proline biosynthetic process (GO:0006561) and proline metabolic process (GO:0006560) and branched amino acid transport (GO:0015803).

In addition, due to the limited-nutrient content in GSPG, the most relevant processes associated with down-regulated genes were synthesis of RNA, RNA processing (GO:0006396), response to stimulus, cellular response to chemical stimulus (GO:0070887) and negative regulation of proteolysis; secondary metabolism related pathways were not significantly overrepresented.

## 3. Discussion

Transcriptome analysis contributes to the identification of transcript abundance during cellular development and environmental perturbations. It may also contribute to elucidating unknown regulatory patterns. Some transcriptomic studies of *Streptomyces* strains have described gene expression using different mutants [25,36,37,38,39]. In this work, a gene expression analysis of *S. clavuligerus,* grown under different nutrient conditions, was performed. This is the first study focused on analyzing the nutritional effect on *S. clavuligerus* metabolism using a complex soybean-based media, widely used for CA production but as yet unexplored at the level of gene-expression.

Overall, it is clear that the soybean-based media favors CA biosynthesis due to the high content of amino acids known to be precursors of CA biosynthesis [6,12,20,40]. CA, as a secondary metabolite, is non-essential for growth; a reduction in the growth rate was observed past 48 h of cultivation (Figure 2), in both the GSPG and SB- media, which coincides with the onset of secondary metabolism [2,13]. Trends for the production profiles are in agreement with those found in related studies [6,12,20,21]. Likewise, the presence of phosphate, at the end, remained in the margins of restrictive concentration thus favoring antibiotic biosynthesis [21].

Interestingly, we observed that decrease in growth rate, complemented by depletion of the carbon source, induces the necessary nutritional stress for triggering CA biosynthesis. The maximum amount of CA in the SB-M condition was observed at a later time of culture, accompanied by unbranched (dispersed) hyphal morphology (box in Figure 2B), and characterized by separate compartments, not observed in GSPG; this might be the result of programmed cell death processes [41]. The biological function of *Streptomyces* programmed cell death remains unclear although it was reported as a mechanism involved in nutrient generation from primary mycelium (MI) consumption, a kind of cannibalism, by the aerial/sporulating mycelium (secondary mycelium MII). According to Bushell [42], the frequency of branching of the vegetative hyphae is strongly dependent on growth conditions, whereby nutrient-rich conditions as SB-M favors branching while, under nutrient-depleted (GSPG) conditions, branching is reduced and growth is dictated by tip extension, thus favoring the formation of the so-called searching hyphae [43]. Moreover, an increase in culture viscosity was observed throughout the SB-M culture experiments; Kim and Kim (2004) suggested that this could be associated with the high density of extracellular polymers and insoluble substrates [23,44].

According to the transcriptome analysis, up-regulated genes in the SB-M condition were involved mostly in regulatory mechanisms for secondary metabolism, and biosynthetic genes for antibiotic production. Consequently, CA and, speculatively, cephamycin C, were accumulated. The transcriptional regulators *CcaR* and *ClaR* have direct and proven influence on CA biosynthetic genes, showing a greater differential expression. The protein product of the *ccaR* gene, located in the cephamycin C gene cluster, is a positive regulator which encodes for both CA and CephC production in *S. clavuligerus*. *ccaR* controls antibiotic biosynthesis by regulating the transcription of biosynthetic genes, e.g., the *ceaS2* promoter, which encodes for the CA pathway intermediary carboxyethyl-arginine (Figure 1) [45].

*claR*, a LysR transcriptional regulator type located in the CA gene cluster, positively regulates the expression of late genes involved in the clavaminic acid conversion to CA [3]; the biosynthetic and regulatory genes showed higher expression profiles when *S. clavuligerus* was grown in the SB-M medium, which favors CA production. Interestingly, regarding the regulation associated with AraC family regulators, the two genes encoding products of this family (SCLAV_1957 and SCLAV_p1319) were up-regulated along with the transcriptional regulator SCLAV_p0773, all located in the plasmid, which might suggest a complex extrachromosomal regulation in antibiotic production. Although the *S. clavuligerus* pSCL4 mega-plasmid does not contain essential genes for primary metabolism, it is densely packed with a large number of clusters for secondary metabolite production, including ß-lactam antibiotics [46]. In this study, the clavam gene cluster and *cas1* were not significantly expressed; this is consistent with CA production in SB-M and the positive regulation of the paralogous gene, *cas2*. It might indicate that, for this experimental condition, the production of CA and cephamycin C was benefited, rather than the production of clavams.

An alternative regulation mode that commonly controls antibiotic production in *Streptomyces* involves sigma factors and related mechanisms, e.g., anti-sigma and anti-anti-sigma factors [47]. Transcriptional regulation by sigma factors is common in bacteria such as *Bacillus* sp. for the control of sporulation and other responses associated with stress. The genome of *S. clavuligerus* presents 75 sigma factors, which suggest a sigma factor-based regulation. The RNA polymerase sigma factor (SCLAV_2754) and the sigma factor σ^70^ type (SCLAV_p0769) are proposed to be involved in the coordination of transcriptional regulation in response to environmental factors such as nutritional, osmotic and oxidative stress; apparently, they are also associated with development (differentiation), yet currently there is no certainty over their specific function [47].

Furthermore, the sigma factor *orf21*, reported as a regulatory gene, does not show significant changes in expression when CA synthesis is carried out (Figure 3). It is suggested that the *orf21* expression could be influenced by environmental factors, e.g., at the onset of nutrient depletion. In addition, the RNA polymerase containing the primary factor that initiates the transcription of a large number of genes, commonly expressed during the exponential growth phase and at 104 h, is already in its latency phase; therefore, the transcription of certain genes has already ceased [48,49]. Culture conditions along with the sampling time, might explain the results. Further gene expression profiling studies at different cultivation times would contribute to clarify this uncertainty.

In addition to sigma factors, the Lux-R (LTR) or Lys-R regulators represent the most abundant regulators in prokaryotes; these regulate various groups of genes that, for the case of *Streptomyces,* involve antibiotic synthesis [50]. The LAL family, a poorly studied group of transcriptional regulators, some of which have been identified in antibiotic clusters and other secondary metabolites of actinomycetes, have been considered path-specific regulators [51]. In this study, LuxR family transcriptional regulator (SCLAV_4464), located on the chromosome, seems to be favored (3.086 LogFC) for the high antibiotic production condition; the same was observed for the Lys-R family transcriptional regulator SCLAV_p1321 (4.231 LogFC), located in the plasmid, close to the cephamycin C cluster. However, there is no experimental evidence about potential interactions between these regulators and the antibiotic produced by *S. clavuligerus*.

Moreover, the regulatory protein *AdpA* (SCLAV_1957) has been extensively studied in *Streptomyces griseus* and *S. coelicolor* species; it appears to regulate sporulation, morphogenesis and antibiotics production [52]. The assays performed by Lopéz-Garcia et al., 2010, using *S. clavuligerus* in relation to *AdpA*, showed that the deletion of the regulator resulted in a low formation of aerial mycelium and the absence of sporulation itself. This apparently leads to a 10% decrease in the concentration of CA compared to the control strain, as a result of low expression of the regulatory genes *ccaR* and *claR* (7- and 4-fold, respectively) [53]. For the conditions assessed in this work, *ccaR*, *claR* and *adpA* were significantly up-regulated (4.2, 3.39 and 2.83-fold respectively) coinciding with high CA concentration.

The expression of *adpA* exhibited a similar behavior to that found for *ccaR* and *claR*. The higher expression in *claR* relative to *ccaR* could be explained if one considers that the *adpA* regulatory effect on these regulators is more noticeable in relation to CA synthesis than that with cephamycin C. Our results agree with this observation; regulatory cascades for both CA and cephamycin biosynthesis, are apparently different since the expression of the CA cluster showed a slightly higher expression compared to that of CephC [49]. Moreover, soy proteins present in the SB-M medium are a major nitrogen source; soy protein assimilation is mediated by extracellular proteases produced by *Streptomyces* sp. However, when the amino acid source is scarce at the end of the exponential growth phase, protease induction guarantees cell development. Different metalloproteases have been identified in species such as *Streptomyces lividans*, *Streptomyces cacoi*, *Streptomyces fradiae* and *Streptomyces griseus,* playing a fundamental role in mycelial growth and cell viability [52]. In *Streptomyces griseus*, it was found that the *adpA* regulatory cascade, besides controlling antibiotic production of streptomycin (strR regulator), was associated with metalloendopeptidase enzyme synthesis (*sgmA*) to cannibalize the primary mycelium (MI) [54]. *sgmA* encodes a zinc-dependent metalloprotease; its deletion causes an aerial mycelium formation delay. In deletion mutants of *adpA*, *sgmA* transcripts were not detected, indicating that *AdpA* serves as a transcriptional activator of *sgmA* [54]. Further, since *AdpA* acts as a switch for aerial mycelium formation, it may also be involved in hydrolytic enzyme expression (lipases, nucleases and proteases) whose action allows cellular differentiation by degradation of cytoplasmic contents, causing hyphal dispersion, followed by secondary mycelium formation and ending with the cellular death phase [37].

As expected, our results suggest that the soy-based culture medium components induce the formation of extracellular hydrolases and proteases in *S. clavuligerus* (Table 1), forcing the bacterium to further degrade the protein during growth, thus ensuring a constant supply of nutrients, essential for primary and secondary metabolism. Within the up-regulated genes we found two metalloproteases (SCLAV_4112 and SCLAV_4359), which are strongly influenced by the nitrogen source. It is known that, in batch cultures, the production of extracellular proteases occurs at low or intermediate growth rates; yet, when the bacterium presents high growth rates the post-exponential phase is delayed, suggesting that formation of proteases can be induced by the decrease of amino acids present in the medium [55]. In the genus *Bacillus*, the formation of protease and sporulation are often triggered by a nutritional deficit, and by genes that have pleiotropic effects on such processes [56]. Nonetheless, the relationship of proteases and the initiation of morphological differentiation in *Streptomyces* species other than *S. griseus* has not been well established [57].

Some authors have suggested that the *adpA* regulator found in *S. clavuligerus* is involved both in antibiotic production and morphological differentiation, similarly to *S. griseus* [53]. The zinc-dependent metalloprotease, SCLAV_4359, was also found overexpressed in the transcriptional study [36]; the higher expression of the metalloprotease SCLAV_4359 at 104 h at the onset of cell lysis, might suggest that synthesis of this hydrolytic enzyme may be involved in both apoptosis and morphological differentiation, similar to *sgmA* in *S. griseus*.

The *S. clavuligerus* metalloprotease has 681 amino acids and its active site is located in the same region of SgmA (351 and 370, respectively); the similarity between SCLAV_4359 and *sgmA* found by alignment of their amino acid sequences using Blastp [58] was 79%. Until now, studies on proteases in *S. clavuligerus* have been limited compared to other streptomycetes species; it is worthy to consider a further study on SCLAV_4359 as a possible hydrolytic enzyme and its relationship with morphological differentiation. Recently, Álvarez-Álvarez et al., (2014) using a microarray technique, showed that SCLAV_4359 had high expression at the end of stationary phase in a *ccaR* deletion mutant (*Δccar::tsr:*) compared with the reference strain (ATCC 27064); although a logical decrease of CA was evidenced, the SCLAV_4359 expression value was associated neither with morphological development nor with global regulation [36].

Regarding the SCLAV_4359 gene (neutral zinc metalloprotease), it was possible to establish that CA synthesis occurred under conditions of stress induced by exhaustion of the amino acid content, which indicates that nutritional stress, both by nitrogen and carbon, might orchestrate the synthesis of antibiotics by secondary metabolism induction. The potential biological interaction between *adpA,* encoding a regulator, and a neutral-zinc metalloprotease encoding SCLAV_4359, both up-regulated in the SB-M condition, has not been elucidated yet. This identified novel relationship might lead to new insights about metabolic and/or regulatory mechanisms that associate morphological development and antibiotic biosynthesis. These findings strengthen the usefulness and significance of transcriptome analyses in cell metabolic and regulatory studies.

Finally, down-regulated genes were grouped into those necessary for cell maintenance and membrane transport. In this respect, the penicillin-binding proteins PBP (SCLAV_3942 and SCLAV_1087, both located in the chromosome) were differentially expressed; recent reports have shown that, in *S. clavuligerus,* these genes can be expressed during different developmental stages, mainly for peptidoglycan biosynthesis. It is known that there is a possible interference of peptidoglycan synthesis when ß-lactam compounds are produced at high concentrations. However, for the case of the GSPG, CA levels are not that high compared with those observed in SB-M [21]. Regarding regulatory genes, the TetR-family transcriptional regulator, encoded by SCLAV_2302, is a protein involved in the transcriptional control of multidrug efflux pumps, antibiotic biosynthetic pathways, response to osmotic stress and toxic chemicals, catabolic pathway control and differentiation processes [59]. Other transcriptional regulators, encoded by SCLAV_4386, SCLAV_5443, SCLAV_4386, SCLAV_5443, SCLAV_p0423, SCLAV_p1581, and SCLAV_5442, have no evidence of specific regulation.

In conclusion, in this work, it was possible to identify clear differences in gene expression when *S. clavuligerus* is exposed to favorable and unfavorable nutrient conditions. Considering the differences in *S. clavuligerus* morphology during its life cycle, and its metabolic response associated with the environment and nutrient content, found in this work, it is worthy to further study the dynamics of gene expression during these extreme conditions so as to contribute to the understanding of how environmental perturbations may affect genes related to morphological differentiation and secondary metabolism; these topics have not been studied in depth in *S. clavuligerus,* using a systems approach [60].

## 4. Materials and Methods

### 4.1. Bacterial Strain and Cultivation

*S. clavuligerus* ATCC 27064 was used throughout this study. Mycelium was obtained following the standard protocol for *Streptomyces* sp. [61], and was stored in 1.5 mL Eppendorf tubes with a 20% glycerol sterile solution at −80 °C. The seed medium consisted of 50 mL TSB medium (30 g·L^−1^; pH 7.0) in 250 mL baffled Erlenmeyer flasks inoculated with mycelium stock and incubated at 28 °C for 36 h at 220 rpm. The following culture media were used: GSPG medium composed of (g/L): glycerol, 15; sucrose, 20; glutamic acid, 1.5; L-proline, 2.5; NaCl, 5.0; K_2_HPO_4_, 2.0; MgSO_4_·7H_2_O, 1.0; CaCl_2_, 0.4; MnCl_2_·4H_2_O, 0.1; FeCl_3_·6H_2_O, 0.1; ZnCl_2_, 0.05; MOPS (3-(N-morpholino) propanesulfonic acid), 20.9 [62]. The favorable nutrient isolated soybean-based medium (SB-M) (Bell Chem International S.A), composed of (g/L): glycerol, 15.0; soy protein isolate, 10.0; malt extract, 10.0; yeast extract, 1.0; K_2_HPO_4_, 2.5; MgSO_4_·7H_2_O, 0.75; MnCl_2_·4H_2_O, 0.001; FeSO_4_·7H_2_O, 0.001; ZnSO_4_·7H_2_O, 0.001; MOPS, 21.0 [21]. All cultures were performed in 250-baffled (4 baffles) Erlenmeyer flasks containing 50 mL of medium. Flasks were inoculated with seed medium (10% v/v) as described above, at an average initial biomass concentration of 0.09 ± 0.015 g/L. Cultures were incubated at 220 rpm and 28 °C for 120 h.

### 4.2. Analytical Techniques

Culture samples were centrifuged at 14,000× *g* for 10 min at 4 °C, and filtered through a 0.22 µm membrane. CA was determined by HPLC Agilent 1200 (Agilent Technologies, Waldbrom, Germany) equipped with a Diode Array Detector (Agilent Technologies, Palo Alto, CA, USA) at 312 nm, using a reverse phase ZORBAX Eclipse XDB-C_18_ (4.6 × 150 mm, 18μm Agilent Technologies, Palo Alto, CA, USA) column; 96% v/v KH_2_PO_4_ (50 mM, pH 3,2) and a 6% v/v methanol was used as mobile phase at 1 mL/min. CA was imidazole-derivatized at a ratio 1:3; the reaction was kept at 30 °C for 30 min [63]. Phosphate quantification was carried out as molybdivanadophosphoric acid by a colorimetric technique, as described elsewhere [64]. Glycerol concentration was determined based on acidic periodate oxidation of alditols to produce formaldehyde, in the presence of L-rhamnose, and the addition of the Nash reagent; it was spectrophotometrically quantified at 412 nm, as described elsewhere [65]. Due to the presence of suspended particles, mainly in SB-M, the Burton’s method, based on DNA determination, was employed for cell growth quantification [66]. The DNA concentration was determined by the diphenylamine reaction: in this assay, DNA was first hydrolyzed under hot acidic conditions, and the deoxyriboses were further oxidized into 5-hydroxy-4-oxopentanals, which then were dimerized and condensed with diphenylamine; the final products were spectrophotometrically detected at 595 nm [66].

### 4.3. RNA Extraction, Library Preparation, and Sequencing

*S. clavuligerus* was cultured in the GSPG- and SB- culture media as previously described. In order to study the transcripts associated with enzymes related to antibiotic synthesis and the overall transcriptome response to nutrient perturbations, CA concentration, nutrient consumption and growth were quantified over time. RNA extraction was performed during idiophase at 104 h when CA concentration was the highest for the conditions evaluated. Samples were centrifuged at 10,000× *g* for 15 min, and cell pellets were immediately frozen using liquid nitrogen, then stored at −80 °C for subsequent RNA isolation. Total RNA was isolated using Trizol® (Invitrogen®), following the manufacturer’s protocols. All RNA preparations were treated with RNase-free DNase (Promega®) to eliminate genomic DNA contamination; the purity, integrity and concentration of RNA was determined using a Bioanalyzer 2100 (Agilent Technologies®). The total RNA remaining in the supernatant was recovered by ethanol precipitation and quantified by a Bioanalyzer 2100. Only samples with high-quality RNA (RNA integrity number ≥ 7.0) were used in the following mRNA library preparation for sequencing. A cDNA library was constructed and sequenced by Illumina Hiseq^TM^ 2000 (Illumina, San Diego, CA, USA) using the standard Illumina RNA-Seq protocol with a read length of 2 × 100 bases [67].

### 4.4. Reads Mapping to Reference Genome and Differential Expression Analysis

The raw sequencing reads were trimmed for low-quality ends by removing adapter sequences and ambiguous nucleotides, using the software FastQC (version 0.11.3) [68]. All sequencing reads with quality scores (*phred* score) less than 30 (medium quality sequence) and read length below 75 bp (short read length sequence) were excluded using the FastX Toolkit (version 0.0.13) [69]. The remaining high-quality reads were used in subsequent alignments.

The RNA-Seq paired-end reads were mapped using Bowtie2 [70] against the *Streptomyces clavuligerus* ATCC27064 reference genome sequence, available in Genbank (accession number NZ_CM000913.1 and NZ_CM000914.1, chromosome and megaplasmid, respectively) [46]. Prior to alignment, the read counts for each gene were extracted using the *htseq-count* command of HTseq (version 0.6.1) [71] based on all annotated genomic features of the *S. clavuligerus* reference genome (NCBI genome assembly id: 280082) and aligned reads. The total mapped reads and overall alignment rates were 23,186,844 (99.90%) and 19,580,887 (99.87%) in SB-M and GSPG, respectively.

The EdgeR package (version 3.22.3) was used to identify differential gene expression [72], including false discovery rate (FDR) calculations. The total mapped reads for each transcript from HTseq were transformed to *cpm* (counts-per-million read); analyses performed in this study used a log_2_ scale of the data; pseudo-counts of 1 were added to the dataset. The filtering was made using *cpm* as criteria and normalized using the implemented *calcNormFactors* function within EdgeR to find a set of scaling factors for library sizes that minimize the log-fold changes (log FC) between the samples for most genes. Due to the treatment conditions and the typical variations, a negative binomial model was used so as to establish the biological coefficient of variation (BCV); that is, the relative abundance of each gene among RNA samples, associated with biological causes and measurement errors, intrinsic to the sequencing technology [72]. Once the negative binomial model was fitted and the dispersion determined, we proceeded with the *extacttest* procedure for determining differential expression; only values with an associated *p* < 0.05 and FDR ≤ 0.05 were considered to be significant.

### 4.5. RNA-seq Raw Sequencing Data Accession Information

The RNA-seq raw sequencing data of *S. clavuligerus* was deposited in the SRA database of NCBI with accession number SAMN11046362.

### 4.6. Validation of Differentially Expressed Genes by Quantitative qRT-PCR

RNA templates for qRT-PCR were isolated using the UltraClean® Microbial RNA Isolation Kit (MO BIO Laboratories, Inc. CA). The selected genes were: The regulatory protein *AdpA* (SCLAV_1957), the sigma factor *orf21*, present in the CA biosynthesis cluster, the *claR* pathway-specific regulator, and the *ccaR* transcriptional regulator of cephamycin C and CA. Also, for validating differential expression, the metalloprotease SCLAV_4359 was included. As a housekeeping gene, the chromosomal gene *hrdB* was chosen as an internal control and referenced as a constitutively expressed gene [38]. All primers used for qRT-PCR were designed using the Primer3 software [73] and listed in Table S2 (Supplementary Material); the melting temperature was 60 °C, length of 20 nt, and amplicon length of 100 bp. Each qRT-PCR reaction (20 μL) contained: 1.0 μL of 25 ng/μL RNA, 1.0 μL of 10 μM of each forward and reverse primers, and 18 μL of master mix QuantiNova SYBR Green RT-PCR Kit (Qiagen®). qRT-PCR reactions were carried out in a StepOne PCR machine (Rotor-Gene Q 5plex HRM Platform) with the following reaction parameters: 10 min RT step at 50 °C; 2 min PCR initial heat activation at 95 °C, 40 two-step amplification cycles with 5 s denaturation at 95 °C and 10 s annealing and extension at 60 °C; a final 15 min dissociation stage was used to generate a melting curve as well as well as verifying specificity of amplification products. Expression analysis and quantification were performed using the 2^−ΔΔCt^ method (where C_T_ is the cycling threshold); negative controls were carried out to confirm the absence of DNA contamination [74].

### 4.7. GO Term Enrichment Analysis of Differentially Expressed Genes

To identify overrepresented gene ontology (GO) terms in the set of differentially expressed genes, statistical GO enrichment analysis was performed using Ontologizer 2.0 [75]. GO annotations were taken from the annotated reference Uniprot proteome (UPID: UP000002357 accessed by Aug. 2018). Analysis was performed using the parent–child intersection method of Ontologizer followed by Benjamini–Hochberg multiple testing corrections. The “study set” corresponded to the frequency of GO terms in the differentially expressed gene set, while the “population set” indicated the whole set of proteins annotated with GO.

## Figures and Tables

**Table 1:** Differentially expressed genes involved in primary, secondary metabolism and regulatory genes.

Gene ID	Protein Name	log FC	*p*-Value	FDR
**Up-regulated genes in SB-M condition**				
Clavulanic acid biosynthesis				
SCLAV_4194	Clavaminate synthase 2 (*cas2*)	4.388	7.210 × 10^−9^	1.090 × 10^−6^
SCLAV_4181	N-glycyl-clavaminic acid synthetase (*gcas*)	3.694	6.070 × 10^−7^	4.890 × 10^−5^
SCLAV_4191	Transcriptional activator (*claR*)	3.393	2.960 × 10^−5^	1.307 × 10^−3^
SCLAV_4195	Proclavaminate amidinohydrolase (*pah*)	3.303	5.560 × 10^−6^	3.266 × 10^−4^
SCLAV_4196	Carboxyethyl-arginine beta-lactam-synthase (*bls*)	2.850	1.975 × 10^−4^	6.227 × 10^−3^
SCLAV_4190	Clavaldehyde dehydrogenase (*car*)	2.739	5.920 × 10^−5^	2.268 × 10^−3^
SCLAV_4197	Carboxyethylarginine synthase (*ceas*)	2.556	1.492 × 10^−4^	4.988 × 10^−3^
Cephamycin C biosynthesis				
SCLAV_4206	Deacetoxycephalosporin C hydroxylase (*cefF*)	5.875	1.030 × 10^−11^	3.460 × 10^−9^
SCLAV_4208	Cephalosporin hydroxylase (*cmcI*)	5.400	6.900 × 10^−12^	2.610 × 10^−9^
SCLAV_4211	Deacetoxycephalosporin C synthetase (DAOC)	5.104	3.480 × 10^−11^	1.050 × 10^−8^
SCLAV_4205	3′-hydroxymethylcephem-O-carbamoyltransferase (*cmcH*)	4.947	2.180 × 10^−10^	5.280 × 10^−8^
SCLAV_4204	Positive regulator (*ccaR*)	4.226	5.870 × 10^−9^	9.330 × 10^−7^
SCLAV_4199	Isopenicillin N synthetase (IPNS)	4.106	1.050 × 10^−8^	1.550 × 10^−6^
SCLAV_4201	L-lysine-epsilon aminotransferase (*lat*)	4.015	3.810 × 10^−7^	3.390 × 10^−5^
SCLAV_4207	7-alpha-cephem-methoxylase P8 chain (*cmcJ*)	3.274	1.060 × 10^−5^	5.652 × 10^−4^
SCLAV_4200	N-5-amino-5-carboxypentanoyl-L-cysteinyl-D-valine synthase (*pcbAB*)	3.504	5.750 × 10^−7^	4.710 × 10^−5^
SCLAV_4210	Isopenicillin N epimerase (*cefD*)	3.225	5.050 × 10^−5^	2.031 × 10^−3^
Miscellaneous genes				
SCLAV_4359	Neutral zinc metalloprotease	8.313	1.630 × 10^−19^	9.840 × 10^−16^
SCLAV_4112	Extracellular small neutral protease	6.589	6.580 × 10^−14^	6.640 × 10^−11^
SCLAV_4455	Beta-lactamase inhibitory protein	5.316	5.960 × 10^−10^	1.240 × 10^−7^
SCLAV_4202	BLP (Beta-Lactamase Inhibitory Protein)	5.227	2.420 × 10^−10^	5.640 × 10^−8^
SCLAV_p1319	Putative transcriptional regulator AraC family	5.113	2.340 × 10^−9^	4.080 × 10^−7^
SCLAV_2754	RNA polymerase sigma factor	4.943	5.760 × 10^−5^	2.247 × 10^−3^
SCLAV_p1007	Beta-lactamase domain protein	4.755	2.820 × 10^−9^	4.740 × 10^−7^
SCLAV_4723	Beta-lactamase inhibitory protein	4.694	1.720 × 10^−8^	2.480 × 10^−6^
SCLAV_p0769	Sigma factor, σ^70^ type, group 4	4.637	2.243 × 10^−4^	6.856 × 10^−3^
SCLAV_3577	NADH-quinone oxidoreductase subunit N	4.603	6.800 × 10^−6^	7.350 × 10^−6^
SCLAV_p1321	LysR family transcriptional regulator	4.238	1.840 × 10^−7^	1.790 × 10^−5^
SCLAV_4189	Cytochrome P450-SU2 (*cyp*)	3.523	5.630 × 10^−6^	3.277 × 10^−4^
SCLAV_4464	Transcriptional regulator, LuxR family	3.086	4.304 × 10^−4^	1.160 × 10^−2^
SCLAV_1957	AraC family transcriptional regulator (*adpA*)	2.835	3.971 × 10^−4^	1.108 × 10^−2^
Carbon metabolism				
SCLAV_5677	Aldehyde dehydrogenase	5.151	9.470 × 10^−12^	3.370 × 10^−9^
SCLAV_0665	Acyl-CoA dehydrogenase	2.943	2.290 × 10^−4^	6.964 × 10^−3^
SCLAV_3742	Malate dehydrogenase	2.146	1.345 × 10^−3^	2.800 × 10^−2^
SCLAV_1401	Pyruvate dehydrogenase E1 component	2.107	1.630 × 10^−3^	3.202 × 10^−2^
Nitrogen metabolism				
SCLAV_2388	N-acetylglutamate synthase	3.786	2.454 × 10^−3^	4.380 × 10^−2^
SCLAV_0800	Arginine biosynthesis bifunctional protein (*argJ*)	3.184	1.391 × 10^−3^	2.844 × 10^−2^
SCLAV_0796	Argininosuccinate synthase	3.171	8.450 × 10^−6^	4.685 × 10^−4^
SCLAV_4534	Ammonium transporter	2.896	2.420 × 10^−5^	1.087 × 10^−3^
SCLAV_0798	Acetylornithine aminotransferase (*oat2*)	2.582	5.623 × 10^−4^	1.418 × 10^−2^
SCLAV_0799	Acetylglutamate kinase	2.553	1.598 × 10^−3^	3.160 × 10^−2^
**Down-regulated genes in GSPG**				
SCLAV_5442	Putative transcriptional regulator	−6.728	2.427 × 10^−3^	4.380 × 10^−2^
SCLAV_1087	Putative penicillin-binding protein	−4.02	1.310 × 10^−4^	4.536 × 10^−3^
SCLAV_2302	TetR-family transcriptional regulator	−3.962	1.670 × 10^−4^	5.440 × 10^−3^
SCLAV_4124	Putative PadR-like family transcriptional regulator	−3.839	1.910 × 10^−5^	9.100 × 10^−4^
SCLAV_5441	Pyruvate phosphate dikinase	−3.701	5.920 × 10^−4^	1.469 × 10^−2^
SCLAV_3293	RNA polymerase ECF-subfamily sigma factor	−3.549	1.024 × 10^−3^	2.237 × 10^−2^
SCLAV_3942	Penicillin-binding protein	−3.525	4.230 × 10^−4^	1.155 × 10^−2^
SCLAV_1943	Acetyl/propionyl CoA carboxylase	−3.410	2.440 × 10^−5^	1.086 × 10^−3^
SCLAV_3597	Two-component system sensor kinase	−3.380	2.453 × 10^−3^	4.380 × 10^−2^
SCLAV_p0423	Transcriptional regulator, XRE family protein	−3.266	1.760 × 10^−5^	8.660 × 10^−4^
SCLAV_5443	Transcriptional regulator	−2.982	2.360 × 10^−4^	7.104 × 10^−3^
SCLAV_p1581	Transcriptional regulator, BadM/Rrf2 family	−2.875	4.930 × 10^−4^	1.272 × 10^−2^
SCLAV_0603	Putative PadR-like family transcriptional regulator	−2.718	1.960 × 10^−4^	6.213v10^−3^
SCLAV_2046	4-hydroxyphenylpyruvate dioxygenase	−2.707	1.520 × 10^−4^	5.053 × 10^−3^
SCLAV_2979	Putative dihydrolipoamide acyltransferase	−2.679	8.140 × 10^−4^	1.873 × 10^−2^
SCLAV_2974	3-hydroxybutyryl-CoA dehydrogenase	−2.659	4.310 × 10^−4^	1.160 × 10^−2^
SCLAV_4588	Aldehyde dehydrogenase	−2.492	2.513 × 10^−3^	4.447 × 10^−2^
SCLAV_4386	Putative transcriptional regulator	−2.254	9.460 × 10^−4^	2.113 × 10^−2^

## Supplementary Materials

The following are available online at https://www.mdpi.com/2079-6382/8/3/96/s1, Supplementary Material containing Table S1: Differential Gene Expression of *Streptomyces clavuligerus*, Table S2: Oligonucleotides used in this work.
